# Biological effective dose as a predictor of local tumor control in stereotactic radiosurgery treated parasellar meningioma patients

**DOI:** 10.1007/s11060-024-04804-1

**Published:** 2024-08-27

**Authors:** Ahmed Shaaban, Duy Pham, Salem M. Tos, Georgios Mantziaris, David Schlesinger, Jason P. Sheehan

**Affiliations:** Department of Neurological Surgery, University of Virginia, Box 800212, Charlottesville, VA 22908 USA

**Keywords:** Biological effective dose, Para-sellar meningioma, Stereotactic radiosurgery

## Abstract

**Introduction:**

The radio-surgical literature increasingly uses biological effective dose (BED) as a replacement for absorbed dose to analyze outcome of stereotactic radiosurgery (SRS). There are as yet no studies which specifically investigate the association of BED to local tumor control in para-sellar meningioma.

**Methods:**

we did a retrospective analysis of patients underwent stereotactic radiosurgery (SRS) for para-sellar meningioma during the period of 1995–2022. Demographic, clinical, SRS parameters, and outcome data were collected. The target margin BED with and without a model for sub-lethal repair was calculated, as well as a ratio of BED at the target margin to the absorbed dose at the target margin. Factors related to local control were further analyzed.

**Results:**

The study was comprised of 91 patients, 20 (22.0%) and 71 (78.0%) of whom were male and female, respectively. The median age was 55.0 (interquartile range Q1, Q3:47.5,65.5years). 34 (37%) patients had a resection of their meningioma prior to SRS. The median interval from SRS to last clinical follow up or progression was 89 months. 13 (14.3%) patients were found to have progression. 3-, 5- and 10-years local tumor control were 98%, 92% and 77%, respectively. In cox univariate analysis, the following factors were significant: Number of prior surgical resections (Hazard Ratio [HR] = 1.82, 95% CI = 1.08–3.05, *p* = 0.024), BED (HR = 0.96, 95% CI = 0.92-1.00, *p* = 0.03), and BED/margin (HR = 0.44, 95% CI = 0.21–0.92, *p* = 0.028). A BED threshold above 68 Gy was associated significantly with tumor control (*P* = 0.04).

**Conclusion:**

BED and BED /margin absorbed dose ratio can be predictors of local control after SRS in parasellar meningioma. Optimizing the BED above 68Gy_2.47_ may afford better long-term tumor control.

## Introduction

The most common treatment planning paradigm for Gamma Knife radiosurgery (GKRS) involves creating a distribution of absorbed radiation dose that will deliver what is thought to be a minimally-effective dose conformally to the surface (often termed margin) of the target [1]. Traditionally, this dose distribution is designed so that the absorbed dose at the margin represents an absorbed dose that is approximately 50% of the maximum absorbed dose within the target (i.e., the dose is prescribed to the 50% isodose line). However, studies have examined the effectiveness of alternative relative margin absorbed doses [1].

For GKRS, the treatment dose distribution is created by applying roughly spherical volumes of radiation (often referred to as “shots”) to the treatment plan, where each shot represents the sum total of all of the beams (usually 192 beams if no beams are blocked) [2]. There is usually no expansion between the observed margin of the target on neuro-imaging and the location of the prescribed absorbed dose level [2, 3]. The radiation dose delivered by each shot combines to create the complete dose distribution to the target. Geometrically-complicated dose distributions can be created by using multiple isocenters with different collimations, locations, and relative weights [3]. It is important to note that in GKRS, the shot-based delivery technique means that the complete tumor is not irradiated to the same dose level at every timepoint of the treatment. The dose rate experienced at given point in the target (and surrounding normal tissue) varies throughout the treatment depending on distance to the shot being delivered and the baseline dose rate of the shot (which in turn depends on the beam sizes comprising the shot and the baseline dose rate of the Gamma Knife’s cobalt sources at that time).

The main biological mechanism through which radiation is thought to affect tissue is through damage to the DNA of the cells being irradiated [4, 5]. Both normal and disease tissue cells have an inherent capacity to repair radiation-induced DNA damage [5, 6]. A variety of biological models have been formulated that attempt to predict this damage [1, 5, 7]. One such model is the biological effective dose (BED) model, which attempts to predict the biological effect of a given absorbed dose, taking into account repair of sublethal DNA damage [7]. Time is often included as a parameter in the BED models to include fast and slow DNA repair mechanisms [7]. BED models have found widespread use in traditional, fractionated radiation therapy [8]. Their use in radiosurgery settings has historically been limited due to difficulties in computing BED values for inhomogeneous dose distributions, the variation of dose rate with time at any given point in the patient, and because there are limitations to the underlying assumptions of the BED models when applied to single- and hypo-fractionated approaches common to GKRS [1, 5, 7].

A recent publication by Hopewell et al. has re-ignited interest in the application of BED models to GKRS by proposing a simplified algorithm for calculating BED at the target margin [7]. The role of BED for outcome prediction has been recently investigated for GKRS for various pathologies such as pituitary adenoma, acromegaly, vestibular schwannoma, intracranial meningioma, brain arteriovenous malformation (AVM) [7, 9–13]. The results of these studies suggest that BED models can provide an explanation for some of the variability of outcomes even when the prescribed absorbed dose to the target margin is uniform across subjects. In our single center study, we investigate the effects of BED on local control in para-sellar meningioma patients treated with stereotactic radiosurgery.

## Methods

### Patient population and inclusion criteria

Patients treated with GKRS at our institution for parasellar meningioma (WHO grade 1 or presumed as grade 1 based on clinical and radiological criteria) between 1995 and 2022 were included in this study. The study was reviewed and approved by our Institutional Review Board (IRB).

Specific inclusion criteria included: (1) Diagnosis of parasellar meningioma based on clinical, radiographical features, or pathological features; (2) Complete clinical data, SRS parameters, outcome details from follow-up; and (3) At least 6 months of follow-up.

Parasellar meningiomas were defined as meningiomas located on one or more of the following areas: cavernous sinus, Meckel’s cave, petro-clival region, optic canal, anterior clinoid process [14].

Clinical information on each patient was collected, including: patient’s demographic characteristics, neurological and endocrinological symptoms pre-SRS, any treatment (i.e., surgical resection or radiotherapy) to the meningioma prior to SRS, SRS dose plan, radiographic, neurological, and endocrinological outcomes at last follow-up, dates of last follow-up, tumor response, and additional treatment modality if needed for tumor progression.

Treatment planning information was collected including the treatment date, prescription dose (i.e., absorbed dose to the tumor margin), maximum dose, tumor volume, number of treatment fractions, maximum dose, number of isocenters, and total beam time.

### Radiosurgery treatment protocol

A detailed description of the method of radio-surgical treatment used can be found in previous studies [15]. Stereotactic frame placement of a Leksell G-Frame was performed in an operating room (OR) setting by a neurosurgeon. An anesthesiologist administered monitored anesthesia to the patient during frame placement. Following frame placement, stereotactic imaging was then obtained for treatment planning. Unless contraindicated, magnetic resonance (MR) imaging was acquired, including pre- and post-contrast 3D T1-weighted pulse sequences (3D MP-RAGE or similar 3D gradient-recalled echo (GRE)). When MR was contraindicated (such as the presence of a cardiac pacemaker), a thin-slice volumetric CT scan was obtained with and without contrast administration.

GKRS dose plans were formulated under the direction of a neurosurgeon in conjunction with a medical physicist and radiation oncologist. The Gamma Knife Model U was used to treat patients until July 2001, Model C from July 2001 to August 2006, the model Perfexion from August 2006-October 2016, and the model Icon from October 2016 to 2022. The Kula treatment planning system was used for dose planning from 1990 to June 1994. Leksell GammaPlan was used for treatment planning from June 1994 to the end of the study in 2022.

### BED calculation

We employed the simplified two-compartment BED model proposed by Jones and Hopewell et al. [16]. This model requires parameters for the radiosensitivity of the tumor (α/β ratio), repair half-lives to characterize slow- and fast-DNA repair mechanisms, the absorbed dose to the target margin, the total beam-time (i.e., the total time the target was irradiated), the number of shots in the treatment, and the length of any inter-treatment pauses in treatment. This last parameter models the shot-by-shot delivery of GKRS. In-between shots, there is a period of time where the Gamma Knife is not actively irradiating the tumor. The length of this inter-shot time varied widely by the model of the Gamma Knife used for the radiosurgery.

For this study, our primary outcome was local tumor control. The values for the above parameters (with the exception of total number of shots, which varies by case) appear in Table 1. Tumor progression was defined as a volumetric increase of ≥ 20% from baseline defined, and a volumetric decrease of ≥ 20% defined as tumor regression. Lesions with a volumetric change less than < 20% from baseline were considered stable [17]. Tumor control was defined as a tumor that either regressed or was stable at last follow up.

**Table 1:** BED calculation parameters

Parameter	Value
Tumor α/β	2.47
Fast repair component half-life (minutes)	16
Slow repair component half-life (minutes)	132
Partition coefficient (relative to fast)	0.49
GK Model U intershot time (minutes)	5.0
GK Model C inter-shot time (minutes)	0.80
GK Model Perfexion/Icon inter-shot time (minutes)	0.2

We calculated a margin BED for each GKRS treatment both with (BED with repair) - and without (BED without repair) including the repair term. We also calculated a ratio of margin BED with repair / margin dose as a method to quantity the magnitude of the difference between BED and margin dose.

### Statistical analysis

Statistical analysis was performed using R language [R foundation of statistical computing V R-4.3.2]. All statistical tests were two-sided, and p values < 0.05 were considered statistically significant. Descriptive statistics for categorical variables were reported as frequency (percentage), and for continuous variables as median (inter quartile range 1st Quartile, 3rd Quartile). Univariate and multivariable logistic regression were used to evaluate the factors related to the overall odds of local failure. Cox regression was used to evaluate the factors related to time to local failure and accounted for subject censoring during the study. For all tests, predictor variables with p-values < 0.10 in univariate analyses were selected for inclusion in the multivariate model.

## Results

### Demographic and radiosurgical parameters

91 patients met our inclusion criteria, 20 (22.0%) and 71 (78.0%) of whom were male and female, respectively. At the time of SRS, the overall cohort had a median age of 55.0 years (47.5, 65.5 years). 89 patients (97.8%) had signs and/or symptoms related to their meningioma. 63 (69.2%) patients had one or more cranial nerve deficits, while 26 (28.6%) presented with headaches. 34 (37%) patients had a resection of their meningioma prior to their initial SRS. The median interval from SRS to last clinical follow up or progression was 89 months (43, 145.5 months). Overall, 13 (14.3%) patients were found to have progression after first radiosurgery.

**Fig. 1:**
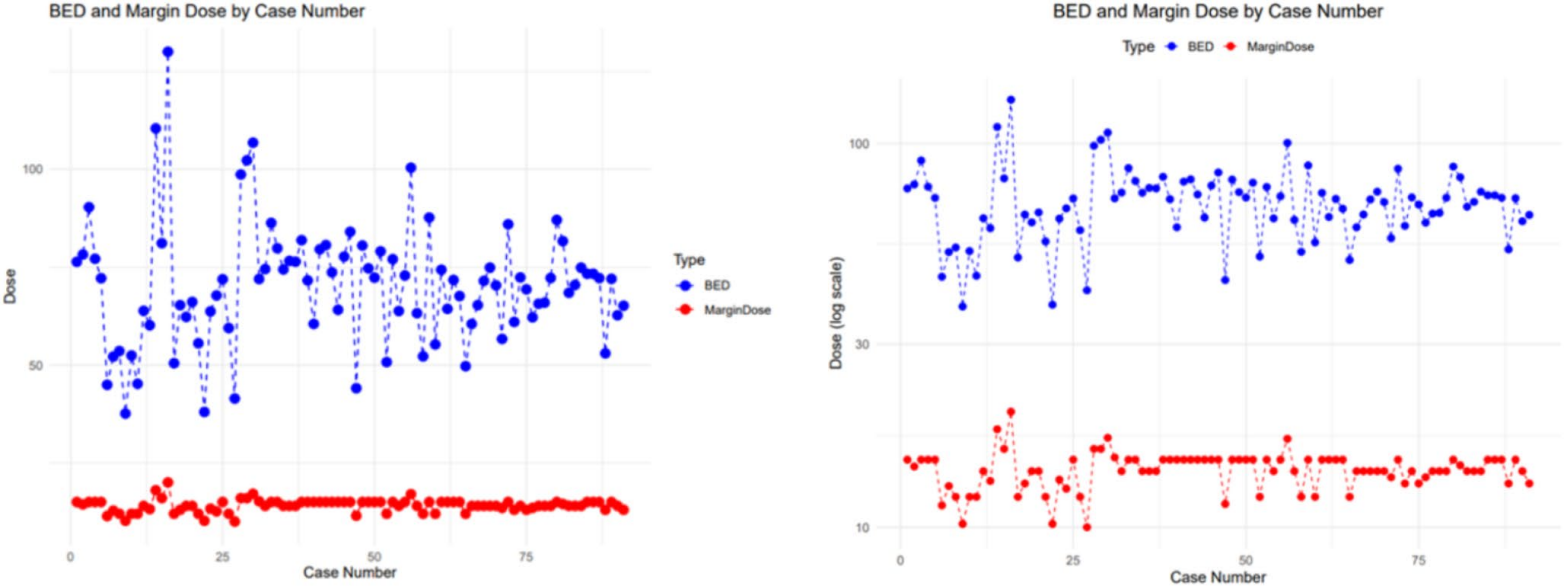
Parallel coordinates plot of margin dose and corresponding BED for each of the 91 cases included in the study

In terms of their SRS treatment parameters, the median margin dose was 14.0 Gy (13, 15) with a median isodose line of 50.0% (37.5, 50) delivered in a single fraction. The median biological effective dose (BED) was 71.6 Gy_2.47_ (62, 77). The tumor volume treated had a median volume of 4.75 cc (2.4, 7.68 cc). The median BED/margin ratio was 4.89(4.5, 5) **(**Tables 2 and 3). Figure 1 shows the relationship between the margin dose and BED for each of the included cases.

**Table 2:** Demographic and clinical characteristics

Factor	Number of patients or Median (% or range)
Demographic factors	
Gender	
Male	20 (22.0)
Female	71 (78.0)
Median age at SRS (range)	55.0 (47.5, 65.5)
Prior resection to SRS	34 (37)
Number of resections prior to SRS	
One	26 (28.6)
Multiple	8 (8.8)
Clinical presentations	
Symptomatic at SRS	89 (97.8)
Hyperprolactinemia	5 (5.5)
ACTH deficiency	1 (1.1)
TSH deficiency	1 (1.1)
Panhypopituitarism	1 (1.1)
Cranial nerves deficit	63 (69.2)
Headache	26 (28.6)
Clinical follow up after SRS or until progression	89(43, 145.5)
Tumor progression	13 (14.3)
Additional treatment after progression	
Repeat SRS	12(13)
Resection	1 (1)
Complications	
No cranial nerve deficit	31(34)
Cranial nerve Same like before SRS	21(23)
Cranial nerve Better after SRS	2(2)
Cranial nerve worse after SRS	2(2)
Newly developed cranial nerve deficit	4(4)
Unknown	31(34)
Endocrinopathy	0

**Table 3:** Treatment parameters of SRS (n = 91)

Factor	Median (range)
Margin dose (Gy)	14.0 (13, 15))
Tumor volume (cc)	4.75 (2.4, 7.68)
Isodose line (%)	50.0 (37.5, 50)
BED (Gy)	71.6 (62, 77)
BED/Margin ratio	4.89 (4.5, 5)

### Tumor control

3-, 5- and 10-years local tumor control were 98% (95%, 100%),92% (85%, 98%) and 77% (66%, 89%) respectively.

**Fig. 2:**
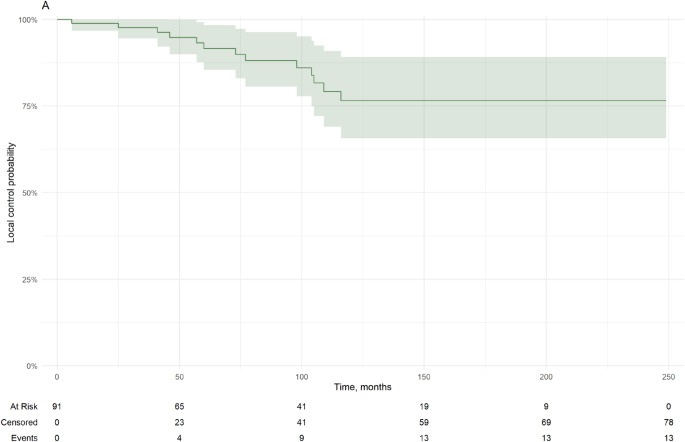
Kaplan Meier of local tumor control for parasellar meningiomas in this series

**Fig. 3:**
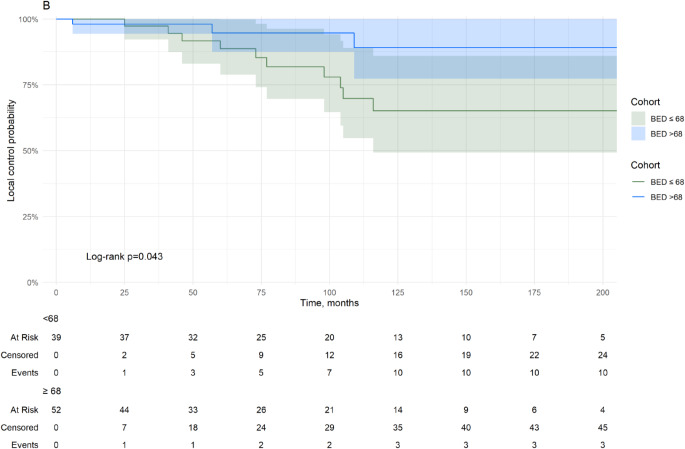
Local tumor control for a BED greater than 68 Gy versus less than or equal to 68 Gy

In Cox univariate analysis, the following factors were significant: Number of prior surgical resections (Hazard Ratio [HR] = 1.82, 95% CI = 1.08–3.05, *p* = 0.024), BED (HR = 0.96, 95% CI = 0.92-1.00, *p* = 0.03), and BED/margin (HR = 0.44, 95% CI = 0.21–0.92, *p* = 0.028). Further details may be found in Table 4; Fig. 2. A BED threshold above 68 Gy_2.47_ was associated significantly with tumor control (*P* = 0.04) at last follow up (Fig. 3). 42.9% (39/91) patients had a BED below 68 Gy_2.47_.

**Table 4:** Univariate / multivariate analysis using cox proportional hazard method

Factor	Univariate		Multivariate *	
	HR (95% CI)	p	HR (95% CI)	P
Age at SRS	1.00 (0.96–1.04)	0.96		
Sex (Ref = Male)	0.45 (0.14–1.47)	0.19		
Prior surgical resection	1.13 (0.38–3.38)	0.82		
Number of prior surgical resections	1.82 (1.08–3.05)	0.024	1.86(0.87, 3.96)	0.11
Interval between resection and SRS (months)	1.00 (0.96–1.03)	0.77		
Recurrence after resection and before SRS	1.21 (0.45–3.27)	0.70		
Types of SRS treatment	0.86 (0.38–1.93)	0.71		
Tumor volume (cc)	0.82 (0.67–1.02)	0.072	0.74(0.47, 1.15)	0.18
Margin dose (Gy)	0.88 (0.65–1.18)	0.39		
Biological effective dose (Gy)	0.96 (0.92-1.00)	0.03		
Biological effective dose/Margin dose	0.44 (0.21–0.92)	0.028	0.28 (0.06, 1.23)	0.092

### Complications

Our observation for cranial nerve (CN) deficit include: 31 (34%) no CN deficit, 21 patients (23%) no change as before SRS, 2 patients (2%) became better after SRS, 2 patients (2%) became worse after SRS, 4 patients (4%) developed new CN deficit after SRS but unfortunately the remaining patients had no data available. Regarding Optic nerve (2 patients got worse and 1 patient newly developed), oculomotor nerve (1 patient became worse and 1 patient newly developed), trochlear nerve (1 patient became worse) trigeminal nerve (newly developed in 4 patients). None of our patients developed endocrinopathy after SRS.(Table 2).

## Discussion

In our series, the overall 3, 5, and 10-year local tumor control rates were 98% (95%, 100%), 92% (85%, 98%), and 77% (66%, 89%) respectively. Our cohort of patients were mainly females as meningioma are more common in females [18].To our knowledge, our series is the first series to specifically relate BED and BED/margin ratio to local tumor control of para-sellar meningioma after stereotactic radiosurgery with a median follow-up of 46 months. We found that both of these variables were significantly associated with local tumor control.

### Stereotactic radiosurgery for parasellar meningioma

Parasellar meningioma are the most common tumors of parasellar region and constitute 15% of all meningioma [19]. A multidisciplinary approach is strongly recommended for meningioma of such a location as gross total resection is usually not feasible without incurring significant morbidity [14, 20]. Management options include watchful observation, open surgical resection, endoscopic resection, or stereotactic radiosurgery (SRS) [14]. With a reported resection associated mortality of 7% and morbidity of 60% for parasellar meningiomas, SRS has become a favored option as a primary, adjuvant, or salvage treatment of parasellar meningioma [15, 21, 22]. Based on a recent International Stereotactic Radiosurgery Society (ISRS) Practice Guideline of cavernous sinus meningiomas, SRS was considered a safe and effective option, with 5-year progression free survival ranging from 86 to 99% and 10 year progression free survival ranging from 69 to 97% [23]. The ISRS guideline recommended margin doses from 11 to 16 Gy (level III evidence) [23]. The margin doses and local control rates in our series are consistent with these results.

However, the small percentage of local failures in both the ISRS review and our current series requires further investigation. Multiple factors have been reported to affect outcome of SRS in parasellar meningioma including: tumor volume, prior surgeries, margin dose, histological grade, progression after microsurgery, Ki-67 index, and extent of resection [24]. For this study, we hypothesized that BED may also be associated with the probability of local control.

### Rationale for investigating biological effective dose (BED)

Fractionated radiation therapy has long relied on the linear-quadratic (LQ) model to describe the relationship between the proportion of cell-kill and absorbed [24]. While the mechanisms underlying the LQ-model remain uncertain [25], as a model it has proven to be a useful mechanism to describe the relative radiosensitivity of different tumors and normal tissue types. The BED concept is derived directly from the LQ-model and is defined as “the total dose required to give the same log cell kill as the schedule being studied, at an infinitely low dose rate or with infinitely small fractions well‑spaced out” [24, 26]. The BED model has been generalized to account for cell-repopulation effects by adding terms to describe repopulation that occurs during and between radiation dose delivery.

However, until recently there has been little interest in investigating the BED concept in the setting of SRS. SRS is conceptually quite different from fractionated radiosurgery in that an ablative dose of radiation is delivered in one or a small number of treatment fractions, and not at infinitely low dose rates [27]. SRS has proven to result in high probability of local control across a variety of indications, yet there are still a small subset of sub-optimal responders, even when all patients are treated with uniform margin doses of radiation [9, 28, 29].

GKRS in particular has time/dose-rate dependencies which could hypothetically impact the radiobiological effectiveness of a treatment. GKRS uses cobalt-60 as a radiation source, which is a radioactive isotope with a half-life of 5.26 years. Thus, in GKRS the dose rate is variable, and treatments which are given the same margin absorbed dose but delivered 5.26 years apart will have dose rates that differ by a factor of two. In addition, the shot-by-shot delivery of a Gamma Knife system means that there are pauses in radiation delivery as the system prepares to move the patient from one stereotactic location to another [30]. The amount of “non-irradiation” time this takes depends on the Gamma Knife system used. For instance, the model U Gamma Knife required a manual procedure to set a stereotactic coordinate, mount the correct tertiary collimator on the unit, and delivery the radiation [31]. This procedure could take many minutes to accomplish. By contrast, the model Icon Gamma Knife currently used at our institution moves the patient and changes collimator settings in a completely automated fashion that takes only seconds [32]. It seems reasonable to hypothesize that the amount of cellular repair of radiation damage could significantly differ between historical and recent Gamma Knife technologies, and this may explain some of the cause for local failures in GKRS for a given margin absorbed dose [9]. While the high doses and small number of fractions that characterize SRS depart from the assumptions of the LQ-model and BED models, there is evidence that demonstrates dose rate may still play an important role in SRS efficacy. For example, irradiation of 10 cm of spinal cord of pigs with two different dose rates (25 Gy in 25 min vs. 140 min) resulted in 0% radiation induced myelopathy in the longer time plan^1^. Simply put, with a longer treatment time, the lower the biological effective dose and the more the repair of sublethal radiation induced changes [1, 9].

There have been several prior attempts to create BED models tailored for GKRS delivery, and prior studies have found mixed results when applying these models to observed clinical outcomes. Millar et al. calculated BED based on an equation including dose rate, inter-isocenter time, exposure time, sublethal radiation damage repair slow and fast rates, total dose [1, 33–35]. Other models were used to calculate BED by Jones and Hopewell [16] as well as Graffeo et al. [29]. Initial studies by Graffeo et al. [29] and Balossier et al. [28] found an association between BED and biochemical remission in acromegaly patients which was statistically significant only in the 2nd study. Dumot et al. concluded that BED is a strong predictor of endocrine remission in acromegalic patients with a cut off value of > 170 Gy_2.47_ [9]. Hopewell et al. found variation of 15% for a physical dose for an individual patient with vestibular schwannoma [7]. The same group suggested that treatment with smaller number of iso-centers and shorter treatment time achieved the highest biological effective dose [7].

Tuleasca et al. found a clear association between BED and the incidence of hypesthesia in trigeminal neuralgia patients with incidence of 42% after approximately 2600 Gy_2.47_ [36]. In another study, Tuleasca et al. showed BED as a strong predictor of obliteration of unruptured AVM after GKRS [13]. In our series, while margin dose was not statistically significant as a predictor of local control, BED and the BED/margin ratio did show a statistically significant association. Huo et al. found significant association between BED > 50 Gy_2.47_ and local control for intracranial meningioma but their series wasn’t specific for para-sellar meningioma [12]. The same group showed that margin dose > 12 Gy did not achieve better control, but it was associated with more incidence of edema and radiation induced changes [12, 37]. In our series, we found a BED threshold of 68Gy_2.47_ was correlated with better tumor control specifically for parasellar meningiomas. As such, optimization of BED at the time of radio-surgical planning for parasellar meningiomas may serve as an avenue for improving long-term tumor control.

### Study limitations

While our study is the first to address specifically relation of BED to parasellar meningioma outcome, we recognize the study has several limitations. Most importantly, our study is retrospective, and it is limited in sample size. This may introduce bias into the results in the form of selective inclusion and limited statistical power.

The assumptions that we used for parameters in the two-compartment BED model each have some uncertainty, Significantly, the α/β ratio for meningioma and slow/fast repair half-lives are estimates from the simplified BED model used in our investigation. The inter-shot times we utilized for each Gamma Knife are estimates from our experience. However, the BED model is fairly insensitive to small differences in these parameters [1, 38].

As mentioned above, the many fraction/low dose rate assumptions of the BED model depart significantly from the way GKRS is delivered. Also, the simplified 2-compartment BED model employed in our study assumes the entire tumor receives a uniform dose (and thus a uniform BED), and that the entire tumor is receiving the same dose/BED whenever radiation is being delivered, rather than in the shot-by-shot method employed during GKRS. The shot-by-shot delivery of the Gamma Knife creates a complicated relationship between a given tumor’s volume and shape and the treatment delivery time, which in turn is related to the BED. This relationship depends on many factors, including the current activity of the Gamma Knife 60Co sources, the number and size of the shots used in the treatment, and the prescribed margin dose. However, several studies have now demonstrated that BED may be significantly associated with outcome, and our study is consistent with those results. Our results showed an association between BED and local control as well as BED normalized to margin dose and local control, while failing to demonstrate an association between margin dose and local control. In addition, there is a colinear relation between margin dose and BED which may affect confidence intervals and significance. However, further investigation using larger datasets and across a wider range of dose rates and BEDs is required to better understand the magnitude of any association.

One practical limitation of our study is that the BED calculation is performed off-line, as there is not currently functionality within GammaPlan to perform real-time BED calculations. While calculation of the simplified model used in this study has negligible calculation time, there is some data collection overhead that makes it unlikely to become a standard clinical practice without further refinement and integrated functionality within GammaPlan.

## Conclusion

BED and BED /margin ratio can be predictors of local control after SRS in para-sellar meningioma. Optimizing BED above 68Gy_2.47_ can result in improved tumor control in para-sellar meningioma patients. BED may be a parameter to consider in SRS planning. More robust evidence is required to confirm our current results.

## Data Availability

No datasets were generated or analysed during the current study.

## Declarations

### Previous presentations

Nil.
